# Posidonia Natural Residues as Growing Substrate Component: An Ecofriendly Method to Improve Nutritional Profile of Brassica Microgreens

**DOI:** 10.3389/fpls.2021.580596

**Published:** 2021-06-24

**Authors:** Massimiliano D’Imperio, Francesco Fabiano Montesano, Nicola Montemurro, Angelo Parente

**Affiliations:** Institute of Sciences of Food Production, CNR – National Research Council of Italy, Bari, Italy

**Keywords:** “organic” biofortification, mineral enrichment, organic waste, alternative substrate, soilless system

## Abstract

The aim of this study was to test *Posidonia oceanica* (L.) Delile seagrass residues (leaves and fibers) as growing media component to improve the nutritional quality of two different brassica microgreens (Mizuna and Rapini). We hypothesized that addition of posidonia residues in the substrate would result in higher concentration of certain mineral nutrients in the edible parts of plants. Substrates were obtained by mixing leaves and fibers, each material at the rate of 25, 50 and 75% (v/v), with a peat based commercial substrate, that was also used at 100% rate as a control treatment. Two experiments were carried out (Experiment 1: Mizuna microgreens production in growth chamber conditions; Experiment 2: Mizuna and Rapini microgreens production in greenhouse conditions). Plant growth measurements and chemical analysis on edible parts (mineral tissue composition and main bioactive compounds - polyphenol, chlorophylls and carotenoids contents) were performed in order to evaluate the effects of the different substrates on growth and nutritional composition of brassica microgreens. In order to evaluate the consumer safety, daily intake, percentage of recommended daily allowance for I (RDA-I) and hazard quotient (HQ) for I intake through consumption of 50 and 100 g portions of Rapini microgreens were calculated. Posidonia in the growing media mixtures increased I and B content in edible parts of microgreens. The calculated HQ underlines the safety of these products. Results confirm the possibility to improve nutritional profile of brassica microgreens by using this natural material as a growing media component, resulting in a sustainable approach.

## Introduction

The agrifood sector tends to adopt and promote sustainable patterns of production and consumption, in order to cope with the increasing needs of the growing world population and the necessity to increase sustainability. The primary objective of agriculture is to ensure adequate quantities of food, to satisfy nutritional exigencies and to fight nutritional deficiencies, with the effort to minimize negative impacts of production processes on the environment. In this framework, plant foods are considered as a fundamental source of important nutrients, such as vitamins, minerals and bioactive compounds. However, nutritional deficiencies still affect an important part of the world population.

In the last years, several studies were published dealing with different approaches to enhance the nutritional status of target groups of population, thus providing a public health benefit. A promising approach is biofortification. It consists in increasing the content or the bioavailability of nutrients, e.g., vitamins (mainly folate and vitamin A), minerals (calcium, iron, silicon, boron, iodine, zinc, and selenium) and bioactive compounds (polyphenols and carotenoids) in plant foods, in order to ameliorate their nutritional profile in terms of their contribution to satisfy the Recommended Daily Allowance (RDA) for nutrients (White and Broadley, 2009; D’Imperio et al., 2016, 2019, 2020; Montesano et al., 2016; Smoleń et al., 2017; Gonnella et al., 2019). This approach allows improving the nutritional quality of different crops by using several strategies, such as genetic engineering, conventional breeding, and agronomic techniques (Carvalho and Vasconcelos, 2013).

An interesting agronomic technique approach for biofortification consists in the use of soilless cultivation (which includes pure hydroponic or growing media-based systems), allowing for the possibility to act on the nutrient solution composition in order to manipulate at a certain extent the plant nutrients uptake (D’Imperio et al., 2018; Gonnella et al., 2019).

Several Authors suggest that it is possible to improve the nutritional quality of plant food products by taking advantage of natural organic matrices as natural source of essential elements for plant nutrition (Bañuelos et al., 2015; Cervera-Mata et al., 2019), thus minimizing or preventing the use of chemical fertilizers. Such matrices can be selected based on their chemical composition and the related natural endowment of specific plant nutrients, and the consequent possibility to stimulate specific responses in plants aimed to ameliorate their nutritional value. In this context, seagrass and seaweed might represent an important source of mineral elements (Satoh et al., 2019). In the Mediterranean area, the most important seagrass is the posidonia (*Posidonia oceanica* (L.) Del.). Detached parts of plants (residues consisting mostly of leaves that are still almost intact and fibers originated from the deflation of the plant tissues including rhizomes) accumulate in huge amount along the coasts, representing a problem in many coastal sites, with environmental, economic, social and hygienic implications (Cocozza et al., 2011). The composting of posidonia residues allows to obtain high-quality compost materials. It is reported that this compost can be used as soilless growing media component in substitution of peat, leading to important environmental and economic benefits (Montesano et al., 2014; Gattullo et al., 2017). Most researches focused on the use of posidonia residues after composting, while the use of untreated posidonia residues as growing media component is relatively unexplored (Castaldi and Melis, 2004).

Microgreens are gaining an increasing popularity as an innovative horticultural product. Beside the interesting culinary applications as an ingredient to add color and flavor to dishes, microgreens are considered high-nutritional value products. Moreover, microgreens represent a good experimental model to evaluate the effects of innovative growing media, being this typology of vegetables characterized by a very short growing cycle (7–21 days after germination). The commercial production of microgreens is usually performed under controlled environment, inside greenhouse, indoor, or tunnel with different levels of technologies. The main critical aspect in the production of microgreens is the selection of the growing media. This plays an important role in determining the visual and nutritional quality of products (Di Gioia and Santamaria, 2015; Di Gioia et al., 2017).

According to the above considerations, the objective of this study was to test a growing substrate based on *Posidonia oceanica* (L.) Del. in mixture with peat for microgreens production. Specifically, we focused on investigating the effects of increasing rates of posidonia residues in the substrate on plant tissues mineral content. We hypothesized that the addition of raw Posidonia residues in the substrate might represent a natural and renewable source of mineral nutrients able to increase vegetable nutritional quality. Two experiments were performed. In a preliminary test in growth chamber we evaluated the suitability of peat-Posidonia mixtures as growing media at laboratory scale. Then, in a second experiment, we tested the use of such growing media to produce microgreens of two species (Rapini and Mizuna) in real greenhouse cultivation conditions.

## Materials and Methods

### Collection and Preliminary Treatment of *Posidonia oceanica* (L.) Delile Residues

Samples of *Posidonia oceanica* (L.) Delile (PO) residues, both leaves (L) and fibers (F), were collected on a beach in Mola di Bari (BA, Italy), a coastal town in Apulia region (southern Italy, 41°03′,80 N - 17°05′,85 E). After the collection, F and L were washed with rain water (previously collected) in order to remove sand and salt, and successively air dried for 1 week inside a greenhouse at the Experimental farm La Noria of CNR-ISPA (Mola di Bari). The air dried materials were milled (1 mm) and used to prepare the growing substrates under comparison for the production of microgreens, in mixture with a peat-based (50% white peat – 50% black peat) commercial substrate (Brill type 3 special, Agrochimica, Bolzano, Italy), as described in details in section “Experiment 1: Indoor Production of Mizuna Microgreens in Growth Chamber Conditions.” Electrical conductivity (EC) and pH of peat and PO residues (L and F) were analyzed on water-soluble extract (1:5 v/v) according to Mininni et al. (2015). For the measurement of DW, peat and PO residues samples were maintained in a forced draft oven at 105°C until constant weight.

### Experiment 1: Indoor Production of Mizuna Microgreens in Growth Chamber Conditions

The trial was carried out from July 3^rd^ to 22^th^ 2018, in a growth chamber (Sanyo, SGC097.PFX, internal dimension 1200 × 600 × 900 mm, growing height 1300 mm, vertical airflow: 0.2 m/sec) at the Institute of Sciences of Food Production, National Research Council, Italy (ISPA-CNR) in Bari. Mizuna (*Brassica rapa* L.) plants were grown in plastic trays (90 cm^2^, 70 × 120 × 45 mm) filled with seven different mixtures (percentages expressed on a DW basis): (i) CTR (control, 100% peat); (ii) L_25__%_ (75% peat and 25% PO L); (iii) L_50__%_ (50% peat and 50% PO L); (iv) L_75__%_ (25% peat and 75% PO L); (v) F_25__%_ (75% peat and 25% PO F); (vi) F_50__%_ (50% peat and 50% PO F) and (vii) F_75__%_ (25% peat and 75% PO F). EC and pH of each mixture were measured on water-soluble extract (1:5, v:v). Total Porosity (TP), water holding capacity (WC), air capacity (AC) and bulk density (BD) of the mixtures were determined by using a method described by Niedziela and Nelson (1992), chosen because suitable to determine properties as affected by the growing container (plastic trays in our case). The seeds were uniformly distributed on the substrate surface with a density of six seeds per cm^2^. The Mizuna plants were grown at a constant temperature of 20°C and a relative humidity of 80%. During the first three days, lights were kept off to allow seeds germination in the dark. On day 4, the seedlings were exposed to white light (fluorescent tubes color 83 plus 4 incandescent lamps, 40,000 lux 500 μmol m^–2^sec^–1^, 110 W m^–2^). Photoperiod conditions were 12 h dark and 12 h light. The trays were irrigated manually every day using 100 ml of tap water until the germination was completed. After germination, trays were irrigated with a half strength Hoagland nutrient solution (NS). The NS was prepared by mixing macro and micronutrients with distilled water, resulting in a final concentration of (mg/L) 112 N, 117.5 K, 80 Ca, 31 P, 16 S, 12 Mg, 0.135 B, 0.56 Fe, 0.055 Mn, 0.0655 Zn, 0.016 Cu, and 0.025 Mo. A NO_3_-N:NH_4_-N ratio of 84:16 was applied. The NS pH was adjusted to 5.5 – 6.0 using 1 M H_2_SO_4_.

A completely randomized design with four replications and seven treatments was adopted for the study, for a total of twenty-eight experimental units each one represented by a single microgreens growing tray.

### Experiment 2: Production of Mizuna and Rapini Microgreens in Greenhouse Conditions

The trial was carried out from September 17^th^ to October 3^rd^ 2018, in a plastic greenhouse at the experimental farm “La Noria” of the Institute of Sciences of Food Production (ISPA-CNR) in Mola di Bari (BA), southern Italy (41°03′ N, 17°04′ E; 24 m a.s.l.). Plants of Mizuna and Rapini (*Brassica rapa* L.) were grown in plastic trays (90 cm^2^, 70 × 120 × 45 mm) filled with seven different substrate mixtures as reported in Experiment 1 (section “Experiment 1: Indoor Production of Mizuna Microgreens in Growth Chamber Conditions”). The seeds were uniformly distributed on the substrate surface with density of six seeds per cm^2^. During the first three days, the trays were covered in order to allow seeds germination in the dark. On day 4, the seedlings were exposed to natural light. Mean air temperature, relative humidity, and photosynthetically active radiation (PAR) inside the greenhouse during the experiments were: 24°C, 58%, and 211 μmol/m^2^/sec.

The trays were irrigated manually every day using 100 ml of tap water until the germination was complete. After germination, trays were irrigated with a half strength Hoagland NS as reported in the first experiment. A completely randomized design with four replications and seven treatments was adopted for the study, for a total of twenty-eight experimental units each one represented by a single microgreens growing tray.

### Yield and Chemicals Characterization of Microgreens

At the harvest, 22 days after sowing in the first experiment and 16 days in the second experiment, yield [expressed as kg of fresh weight (FW) m^–2^] was evaluated. After weighing, harvested microgreens were maintained in a forced draft oven at 65°C until constant weight for the measurement of DW.

### Extraction and Analysis of the Inorganic Elements

The quantification of inorganic iodine (I) in different samples of peat, PO residues and brassica microgreens was performed by using the protocol described by Gonnella et al. (2019). Briefly, 1 g air dried samples were taken and the I content was extracted with ultrapure H_2_O (Milli-Q Millipore 18 M Ω/cm) at 60°C and stirred for 30 min. After extraction, the samples were allowed to cool down to room temperature. The product extract was well mixed and centrifuged at 10,000 × *g* at room temperature and successively filtered by using 0.45 μm filters (regenerated cellulose, RC). The absorbance of samples was determined at 454 nm, using a UV-1800 spectrophotometer (Perkin- Elmer Lambda 25 spectrophotometer, Boston, MA, United States). The quantification of I in samples was determined by interpolation with a calibration standard curve (0 to 9 μg/L; *R*^2^ = 0.9989).

The Cl, NO_3_, PO_4_ and SO_4_ ions were determined by ion exchange chromatography technique (IC-Dionex DX120, Dionex Corporation, Sunnyvale, CA, United States) with a conductivity detector performed as reported by D’Imperio et al. (2018). Briefly, 0.3 g of DW samples were extracted with solution of Na_2_CO_3_ (3.5 mM) and NaHCO_3_ (1 mM), for 30 min at room temperature. Then, the extracts were diluted and filtered by using 0.45 μm (RC) followed with a Dionex OnGuard IIP (Thermo Scientific) in order to remove organic compounds such as humic acids, phenolic fraction, anthocyanins, tannic acids, lignins and azo dyes from sample matrices. The solutions obtained were analyzed by ion chromatography (IC-Dionex DX120) with a conductivity detector, by using an IonPac AG14 precolumn and an IonPac AS14 separation column (Thermo Scientific) at 35°C, flow 1 mL/min.

The total nitrogen (N_tot_) content was measured only in peat and PO residue (L and F) sample, by using the protocol of Kjeldahl modified by Eastin (1976). After mineralization, the samples were cooled, quantitatively transferred in volumetric flask, diluted, filtered using a 0.45 μm and analyzed with ion specific electrode (Thermo Scientific Orion Star A210 Series). The standards for N analysis ranged from 0.1 to 80 mg/L. The quantification of N_tot_ in the samples was determined by interpolation with a calibration standard curve (*R*^2^ = 0,9974).

For Al, B, Ca, Fe, K, Mg, Na, Mn, Cr, and Zn determinations, 0.3 g samples of peat, PO (L and F) residues and brassica microgreens were digested in a closed-vessel microwave digestion system (MARS 6, CEM Corporation, Matthews, NC, United States) with 10 ml of HNO_3_ (Pure grade, Carlo Erba). The digestion procedure was carried out in two steps: 15 min to reach 200°C and 10 min maintained at 200°C (power set at 900–1050 W; 800 psi). Each solution was diluted to volume with ultrapure H_2_O (Milli-Q Millipore 18 M Ω/cm) and filtered using a 0.45 μm filter. Samples were analyzed with Inductively Coupled Plasma - Optical Emission Spectrometry (ICP-OES; 5100 VDV, Agilent Technologies, Santa Clara, CA, United States) to measure Ca, K, Mg, and Na in radial mode and Al, B, Cr, Mn, Zn, and Fe in axial mode (D’Imperio et al., 2018). In addition, accuracy and precision of chemical analysis (NO_3_, I, Ca, K, Mg, Na, Al, B, Cr, Mn, Zn, and Fe) were evaluated by using two different certified reference materials (CRM): NIST_1573a —tomato leaves and SPIN-1_spinach. The certified and experimental value of CRM are provided in Supplementary Material (Supplementary Table 1). The limits of detection (LOD) and the limit of quantification (LOQ) of the methods were calculated with standard deviation (sd) of the blank (*n* = 10), LOD (sd × 10) and LOQ (sd × 10).

### Extraction and Analysis of Total Polyphenols, Chlorophylls, and Carotenoids

Only for the second experiments the content of total polyphenols was carried out according to the Folin–Ciocalteu method by using the extraction methods reported by D’Imperio et al. (2020). Briefly, 200 mg of lyophilized sample were mixed with 10 mL of solvent mixture (MeOH:H_2_O:CH_3_COOH, 79:20:1% v/v/v). The vials were then placed in a sonicator bath at ambient temperature for 30 min, followed by 1 h in a magnetic stirrer. The mixture was centrifuged at 10,000 × *g* at 4°C for 10 min and the supernatant was transferred into a volumetric tube. The residue was resuspended in 10 mL of MeOH:H_2_O:CH_3_COOH (79:20:1% v/v/v), gently mixed manually, and sonicated for an additional 30 min, followed by stirring (1 h) and centrifugation (10,000 × *g* at 4°C 10 min). The TP content was determined using gallic acid (*R*^2^ = 0.9991) as a calibration standard by using a Perkin–Elmer Lambda 25 spectrophotometer (Boston, MA, United States).

Chlorophylls and total carotenoid content were determined spectrophotometrically, using the extraction procedure reported by Montesano et al. (2018). Briefly, lyophilized samples were homogenized in a fresh solution of 80% acetone (C_3_H_6_O:H_2_O, v/v) and stirred for 24 h at room temperature. After extraction, the samples were diluted and filtered by using 0.45 μm (regenerated cellulose, RC) and the absorbance of the extracts were measured at 662, 645, and 470 nm, using a UV-1800 spectrophotometer (Perkin–Elmer Lambda 25 spectrophotometer, Boston, MA, United States).

### Percentage of Recommended Daily Allowance and Hazard Quotient for Intake of Iodine

The I recommended daily allowance (RDA-I) for children over 12 years and adults is 150 μg (Andersson et al., 2007). Daily intake of I (DI) and percentage of the recommended daily allowance of iodine (% RDA-I) from 50 and 100 g FW of brassica microgreens were calculated. Risk assessment was also conducted by using hazard quotient (HQ) – the risk to human health resulting from the intake of I through consumption of fresh brassica microgreens based on a 70 kg adult. The contribution of iodine from other food sources was not considered. The HQ was calculated according to the Protocol of United States Environmental Protection Agency (IRIS, 2011), using the following equation: HQ = ADD/RFD. ADD is the average daily dose of I (mg of I/kg body weight/day) and RfD is the recommended dietary tolerable upper intake level of I (mg of I/kg body weight/day). The I RfD value for a 70 kg adult is 15.72 μg I/kg/day (1100 μg I/day) as suggest (Kessler, 2009). The ADD for 50 or 100 g portions of brassica microgreens was computed as follows: ADD = (MI × CF × DI)/BW. MI is the I concentration of the brassica microgreens (mg/kg DW), CF is the fresh to DW conversion factor for plant samples (calculated as the ratio of DW to FW; Mizuna indoor production 0.047 on average; Mizuna greenhouse production 0.067 on average and Rapini 0.061 on average), DI is the daily intake of microgreens (kg, taken as 50 or 100 g) and BW is the body weight (kg) of humans, assumed as 70 kg.

### Statistical Analysis

Effects of different treatments were tested using analysis of variance followed by means separation with Fisher’s protected least-significant difference at *P* = 0.05. The statistical software STATISTICA 10.0 (StatSoft, Tulsa, OK, United States) was used for the analysis.

## Results

### Chemical Characteristics and Dry Weight Content of Peat, Leaves and Fibers of Posidonia

The main chemical parameters of peat and PO residues (L and F) are reported in Table 1. In general, the pH and EC values of PO residues were higher than peat, with F showing the highest pH value and L the highest EC value. On the other hand, DM was lower in PO residues compared to peat. Significant differences were found among the materials in terms of mineral contents. On average, the highest Cl and Na contents were found in PO residues (1515 and 5240 mg/kg DW respectively). PO F presented the highest N_tot_ concentration, followed by peat, while PO L showed the lowest value (Table 1). The PO residues showed higher contents of I, B, Mg, Fe, and Zn compared to peat. I and Mg contents were higher in leaf residues than in fibers. The I level in peat material resulted below the limit of quantification (LOQ: 0.1520 μg/l). The B content was much higher in PO residues, with higher values in F, than in peat. The content of Fe and Zn was about 2 and 6 times higher, respectively, in PO residues than in peat, while Mn content was higher in peat followed by L and F (Table 1). Peat and PO L showed similar Al and Ca content (1750 and 40550 mg/kg DW respectively, on average) which were 44 and 47% higher than PO F, respectively. The Cr content in PO and peat was lower respect to LOQ (0.63034 μg/l).

**TABLE 1:** pH, EC (mS/cm), dry matter (DW, mg/100 g FW) and elemental composition (mg/kg DW) of *Posidonia oceanica* (L.) Delile leaves (PO L) and fibers (PO F).

Parameter	Peat	PO L	PO F	Significance
pH	6.11 ± 0.036*c*	9.01 ± 0.014*b*	9.25 ± 0.0458*a*	***
EC	0.385 ± 0.001*c*	1.405 ± 0.001*a*	1.229 ± 0.007*b*	***
DM	40,000 ± 54.6*a*	33,000 ± 1087*c*	37,000 ± 2624*b*	*
N_tot_	3470 ± 176*b*	2760 ± 82*c*	4920 ± 250*a*	***
NO_3_	2840 ± 89.3*a*	1080 ± 29.6*b*	840 ± 29*b*	**
SO_4_	2530 ± 119*a*	1950 ± 45*b*	1710 ± 20*c*	**
Cl	380 ± 17.7*b*	1580 ± 41*a*	1450 ± 28*a*	**
Na	240 ± 9.7*b*	5250 ± 468*a*	5230 ± 243*a*	***
I	< *LOQ*	3.32 ± 0.0368*a*	1.99 ± 0.003*b*	***
Al	1930 ± 321*a*	1570 ± 92*a*	840 ± 18*b*	*
B	10 ± 0.22*c*	2760 ± 124*b*	3380 ± 105*a*	***
Ca	37,800 ± 3038*a*	43,300 ± 2474*a*	19,000 ± 922*b*	***
Fe	2060 ± 185*b*	4670 ± 258*a*	4930 ± 192*a*	***
K	2260 ± 246*a*	1600 ± 89*b*	1090 ± 44*b*	**
Mg	2400 ± 210*c*	7690 ± 385*a*	6150 ± 232*b*	***
Mn	103,688 ± 2257*a*	65,873 ± 3147*b*	40,389 ± 1022*c*	***
Zn	13,506 ± 297*b*	87,216 ± 6734*a*	79,950 ± 3365*a*	***

Data are expressed as mean ± standard error of treatment (n = 3). Significance: ns = not significant; *P ≤ 0.05; **P ≤ 0.01; ***P ≤ 0.001. Means separation within lines by LSD (α = 0.05).

### pH, EC and Main Physical Properties of Growing Media

**FIGURE 1:**
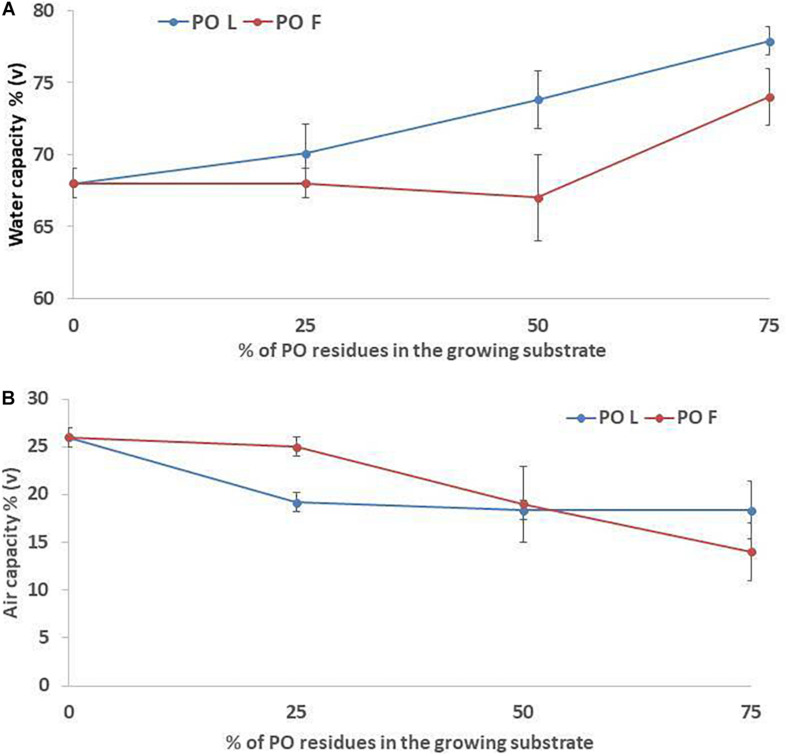
Water **(A)** and air **(B)** capacity values of the growing media mixtures containing *Posidonia oceanica* (L.) Del. Residues (leaves, PO L; fibers, PO F), used for microgreens production. CTR (control 100% peat), L_25__%_ (75% peat,25% PO L), L_50__%_ (50% peat and 50% PO L), L_75__%_ (25% peat and 75% PO L), F_25__%_ (75% peat and 25% PO F), F_50__%_ (50% peat and 50% PO F) and F_75__%_ (25% peat and 75% PO F). Significance: **P ≤ 0.01 (Water capacity); *P ≤ 0.05 (Air capacity).

Increasing PO rate in the growing media mixture resulted in higher pH and EC values, especially when leaves residues were used in the mixture (Table 2). In fact, the highest increase was observed in L_75__%_ treatment (with 16 and 114% of increment, for pH and EC respectively) compared to the control (100% peat). TP was not affected by the addition of PO residues in the mixtures, with a mean value of 92% (data not shown). A slight effect was observed on WC when PO residues, in particular L, were added in the mixtures, although only at the highest rates the effect was significant (*P* = 0.016). The observed values ranged from 68% in the control to 78% in PO L_75_ (Figure 1A). Conversely, AC tended to decrease as an effect of PO residues addiction (p = 0.025). The AC value decrease compared to CTR was observed since a 25% and 50% rate addiction in the case of PO L and PO F, respectively (Figure 1B). BD was 0.10 g/cm^3^ in the CTR, and 0.11, 0.12, and 0.13 g/cm^3^ at 25%, 50% and 75% PO addition rate, respectively, with similar effects of L and F (*P* = 0.001, data not shown).

**TABLE 2:** pH and EC (water-soluble extract 1:5, v:v) values of the growing media mixtures containing *Posidonia oceanica* (L.) Del. Residues (leaves, PO L; fibers, PO F), used for microgreens production.

Treatments	pH	EC (μS/cm)
CTR	6.11 ± 0.02 e	385 ± 1.2 e
L_25__%_	6.70 ± 0.008 d	458 ± 4.09 d
L_50__%_	6.91 ± 0.05 bc	628 ± 8.25 bc
L_75__%_	7.10 ± 0.029 a	826 ± 11.9 a
F_25__%_	6.71 ± 0.008 d	615 ± 14.4 bc
F_50__%_	6.87 ± 0.024 c	660 ± 33.1b
F_75__%_	6.90 ± 0.029 b	604 ± 7.6 c
Significance	***	***

CTR (control 100% peat), L_25%_ (75% peat, 25% PO L), L_50%_ (50% peat and 50% PO L), L_75%_ (25% peat and 75% PO L), F_25%_ (75% peat and 25% PO F), F_50%_ (50% peat and 50% PO F) and F_75%_ (25% peat and 75% PO F). Data are expressed as mean ± standard error of treatment (n = 3). Significance: ***P ≤ 0.001. Means separation within columns by LSD (α = 0.05).

### Experiment 1: Indoor Production of Mizuna Microgreens

**FIGURE 2:**
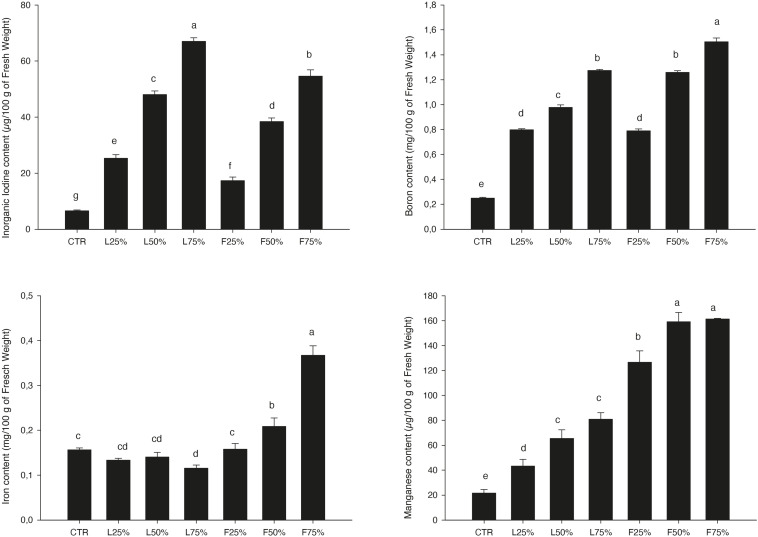
Inorganic iodine, Boron, Iron, and Manganese content in the *Brassica rapa* L. Mizuna as effected by *Posidonia oceanica* (L.) Delile (PO) leaves (L) and fibers (F) in the growing media. CTR (control 100% peat), L_25__%_ (75% peat and 25% PO L), L_50__%_ (50% peat and 50% PO L), L_75__%_ (25% peat and 75% PO L), F_25__%_ (75% peat and 25% PO F), F_50__%_ (50% peat and 50% PO F) and F_75__%_ (25% peat and 75% PO F). Data are expressed as mean ± standard error of treatment (*n* = 3). Significance: ***P ≤ 0.001. Different letters indicate that mean values are significantly different according to the LSD test (α = 0.05).

*Posidonia oceanica* (L.) Delile leaves (PO L) yield was similar to CTR up to a PO residues rate of 50% (in the case of L) and 25% (in the case of F) in the mixture, while generally lower yield values were observed at higher rates (Table 3). Plant DW was not influenced by the treatments: plants accumulated, on average, 4.71 g DW/100 g FW (Table 3). The tissue contents of macro and microelements in Mizuna microgreens were deeply modified by the presence of residues (Table 4). The highest K content was found in Mizuna plants grown in L_75__%_. Plants grown in presence of PO L at rates lower than 75%, as well as plants grown in PO F, showed K content similar to CTR (57.3 g/kg of DW, on average), even if the K content in the PO residues was, on average, lower than peat (Table 1). The increase of PO in growing media led to a clear increase of Mg content in plants. The highest Mg contents were found in L_50__%_, L_75__%_ and F_75__%_. On the contrary, Ca plant tissue concentration was reduced by PO in growing media, compared to peat that showed the highest value. As reported in Table 4, increasing PO (both L and F) in the growing mixture allowed to reduce NO_3_ content in edible parts of plant. Moreover, the addition of PO in growing media modified also SO_4_, PO_4_ and Cl content, with a slight reduction for SO_4_ contents and an increase in PO_4_ and Cl levels. The Na content in Mizuna microgreens produced in PO-based mixtures was higher respect to control, with the highest increase (76%) observed in F_75__%_ treatment (Table 4). Furthermore, the use of PO residues allowed to reduce Al and Cr contents in the microgreens compared to the CTR. The lowest Cr content was found in F_75__%_, while the lowest value of Al was found in the L_75__%_ treatment. A slight increase of Zn content was found only in F_75__%_ treatment. The concentrations of I, B, Fe, and Mn measured in Mizuna plant tissues are reported in Figure 2. The I level in CTR was 6.55 μg/100 g FW, but the plants showed a dramatic increase of I contents as the percentage of PO, both L and F, raised in growing mixture (Figure 2). The highest value of I was found in L_75__%_ (67.06 μg/100 g FW) followed by F_75__%_ (54.61 μg/100 g FW), L_50__%_ (48.0.3 μg/100 g FW) and F_50__%_ (38.41 μg/100 g FW). In the other treatments, values were below 25.5 μg/100 g FW. High increase of B content was also found in Mizuna microgreens produced by adding PO residues in the growing media (Figure 2). The application of PO fibers at 50% and 75% rates increased the Fe content of about 134% respect to CTR (Figure 2). The presence of PO in growing media increased also the Mn contents respect to CTR as reported in Figure 2. The highest Mn contents were found in Mizuna F_75__%_ and F_50__%_ (160.2 μg/100 g of FW, on average) followed by F_25__%_ (126.66 μg/100 g of FW), L_50__%_ and L_75__%_ (73.27 μg/100 g of FW, on average), L_25__%_ (43.38 μg/100 g of FW) and CTR (21.57 μg/100 g of FW). DI, percentage of RDA-I and HQ for the intake of I with 50 and 100 g portions of Mizuna microgreens are reported in Table 5. The application of PO residues in the growing media significantly increased the DI related to 50 and 100 g Mizuna microgreens portions, with higher values obtained when L residues were used (Table 5). Both in the case of 50 and 100 g serving size consumption of L_75__%_ Mizuna microgreens, the RDA-I for adults (150 μg I/day) would not be covered. However, the percentage of RDA-I covered by the consumption of Mizuna microgreens cultivated by using PO residues in growing media was substantially higher than CTR. In addition, the consume of 100 g of serving size of Mizuna microgreens at major content of I was characterized by a HQ value lower than 1, which represents a safe dose (Table 5).

**TABLE 3:** Yield and dry weight (DW) of *Brassica rapa* L. Mizuna group microgreens as effected by *Posidonia oceanica* (L.) Delile (PO) leaves (L) and fibers (F) in the growing media mixtures.

Treatments	Yield	DW
	kg/m^2^	g/100 g FW
CTR	2.40 ± 0.009 ab	4.85 ± 0.03
L_25__%_	2.45 ± 0.12 a	4.64 ± 0.11
L_50__%_	2.37 ± 0.06 ab	4.75 ± 0.056
L_75__%_	1.88 ± 0.13 c	4.78 ± 0.064
F_25__%_	2.10 ± 0.19 abc	4.66 ± 0.09
F_50__%_	1.88 ± 0.13 c	4.62 ± 0.11
F_75__%_	2.06 ± 0.11 bc	4.73 ± 0.03
Significance	**	ns

CTR (control 100% peat), L_25%_ (75% peat and 25% PO L), L_50%_ (50% peat and 50% PO L), L_75%_ (25% peat and 75% PO L), F_25%_ (75% peat and 25% PO F), F_50%_ (50% peat and 50% PO F) and F_75%_ (25% peat and 75% PO F). Data are expressed as mean ± standard error of treatment (n = 4). FW, fresh weight. Significance: ns = not significant; **P ≤ 0.01. Means separation within columns by LSD (α = 0.05).

**TABLE 4:** Mineral composition of *Brassica rapa* L. Mizuna group microgreens as effected by *Posidonia oceanica* (L.) Delile (PO) leaves (L) and fibers (F) in the growing media mixtures.

	K	Ca	Mg	NO_3_	SO_4_	PO_4_	Cl	Na	Cr	Al	Zn
			
Treatments	g/kg of DW			mg/kg of FW				mg/kg DW			
CTR	58.9 ± 1.3bc	30.3 ± 0.21a	4.75 ± 0.05c	4065 ± 48a	1170 ± 27.5a	745 ± 20.4d	518 ± 66e	5850 ± 110e	2.80 ± 0.13a	30.8 ± 1.8a	61.0 ± 0.79b
L_25%_	55.6 ± 1.0c	25.3 ± 0.21b	5.27 ± 0.05b	3332 ± 8.9bc	1101 ± 31.1ab	865 ± 20.3bcd	726 ± 5.7d	8030 ± 131d	2.46 ± 0.23a	24.8 ± 1.3bc	63.9 ± 0.23b
L_50%_	56.6 ± 1.6c	25.1 ± 0.31b	6.00 ± 0.07a	3054 ± 70c	1110 ± 18.1ab	940 ± 21.3abc	775 ± 15.7cd	9964 ± 318ab	1.83 ± 0.07b	27.6 ± 1.3ab	64.3 ± 0.60b
L_75%_	63.1 ± 1.4a	22.7 ± 0.52c	5.83 ± 0.06a	3034 ± 113c	1041 ± 12.4abc	1036 ± 10.8ab	958 ± 46.3ab	9720 ± 145ab	1.46 ± 0.03b	17.1 ± 0.58d	64.5 ± 0.71b
F_25%_	57.5 ± 0.94bc	23.6 ± 0.27c	4.81 ± 0.04c	3535 ± 100b	1013 ± 8.4abc	786 ± 4.6cd	733 ± 38.35d	8871 ± 227c	1.65 ± 0.05b	22.3 ± 1.2c	63.4 ± 0.57b
F_50%_	61.1 ± 1.3ab	23.4 ± 0.46c	5.33 ± 0.05b	3367 ± 41bc	881 ± 12.8bc	764 ± 26.8d	859 ± 20.7bc	9455 ± 261bc	1.42 ± 0.19b	25.3 ± 1.5bc	63.5 ± 0.60b
F_75%_	57.7 ± 0.85bc	22.8 ± 0.68c	5.81 ± 0.08a	3176 ± 32c	810 ± 196.1c	1063 ± 146a	986 ± 9.6a	10310 ± 129a	0.53 ± 0.19c	30.1 ± 0.46a	67.6 ± 1.93a
Significance	***	***	***	***	*	**	***	***	***	***	*

CTR (control 100% peat), L_25%_ (75% peat and 25% PO L), L_50%_ (50% peat and 50% PO L), L_75%_ (25% peat and 75% PO L), F_25%_ (75% peat and 25% PO F), F_50%_ (50% peat and 50% PO F) and F_75%_ (25% peat and 75% PO F). Data are expressed as mean ± standard error of treatment (n = 3). DW, dry weight; FW, fresh weight. Significance: *P ≤ 0.05; **P ≤ 0.01; ***P ≤ 0.001. Means separation within columns by LSD (α = 0.05).

**TABLE 5:** Daily intake, percentage of recommended daily allowance for I (RDA-I) and hazard quotient (HQ) for intake of I through consumption of 50 and 100 g portions of Mizuna microgreens, produced in indoor condition, by adult humans (70 kg body weight).

	100 g Portion of Mizuna microgreens			50 g Portion of Mizuna microgreens		
Treatments	Daily Intake	RDA-I	HQ_100__g_	Daily Intake	RDA-I	HQ_50__g_
	(μg I/day)	(%)		(μg I/day)	(%)	
CTR	6.56 ± 0.35 g	4.37 ± 0.24 g	0.006 ± 0.0002 g	3.28 ± 0.18 g	2.19 ± 0.12 g	0.003 ± 0.0001 g
L_25__%_	25.34 ± 1.3 e	16.90 ± 0.85 e	0.023 ± 0.001 e	12.67 ± 0.64 e	8.45 ± 0.43 e	0.012 ± 0.0006 e
L_50__%_	48.04 ± 1.3 c	32.03 ± 0.91 c	0.043 ± 0.001 c	24.02 ± 0.91 c	16.01 ± 0.45 c	0.022 ± 0.0006 c
L_75__%_	67.07 ± 1.2 a	44.71 ± 0.83 a	0.060 ± 0.001 a	33.53 ± 0.83 a	22.36 ± 0.41 a	0.030 ± 0.0005 a
F_25__%_	17.38 ± 1.3 f	11.59 ± 0.84 f	0.016 ± 0.001 f	8.69 ± 0.84 f	5.79 ± 0.43 f	0.008 ± 0.0006 f
F_50__%_	38.41 ± 1.3 d	25.61 ± 0.86 d	0.036 ± 0.001 d	19.21 ± 0.86 d	12.80 ± 0.43 d	0.018 ± 0.0006 d
F_75__%_	54.61 ± 2.3 b	36.40 ± 1.54 b	0.050 ± 0.002 b	27.30 ± 1.54 b	18.20 ± 0.77 b	0.025 ± 0.001 b
Significance	***	***	***	***	***	***

Data are expressed as mean ± standard error of treatment (n = 3). Significance: ***P ≤ 0.001. Means separation within columns by LSD (α = 0.05).

### Experiment 2: Greenhouse Production

**FIGURE 3:**
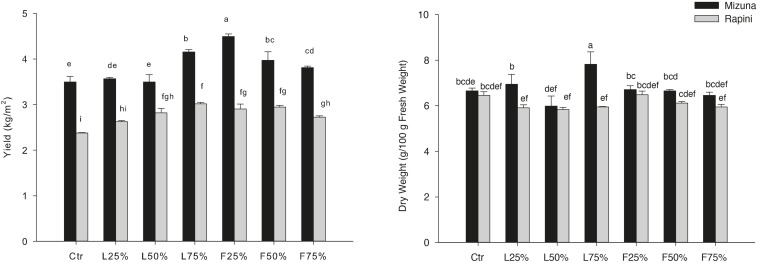
Growing media effect on yield and dry weight (DW) of *Brassica rapa* L. Mizuna and Rapini group microgreens, produced in greenhouse, as effected by *Posidonia oceanica* (L.) Delile (PO) leaves (L) and fibers (F) in the growing media. CTR (control 100% peat), L_25__%_ (75% peat and 25% PO L), L_50__%_ (50% peat and 50% PO L), L_75__%_ (25% peat and 75% PO L), F_25__%_ (75% peat and 25% PO F), F_50__%_ (50% peat and 50% PO F) and F_75__%_ (25% peat and 75% PO F). Significance: *P ≤ 0.05 (DW); ***P ≤ 0.001 (yield). Different letters indicate that mean values are significantly different according to the LSD test (α = 0.05).

**FIGURE 4:**
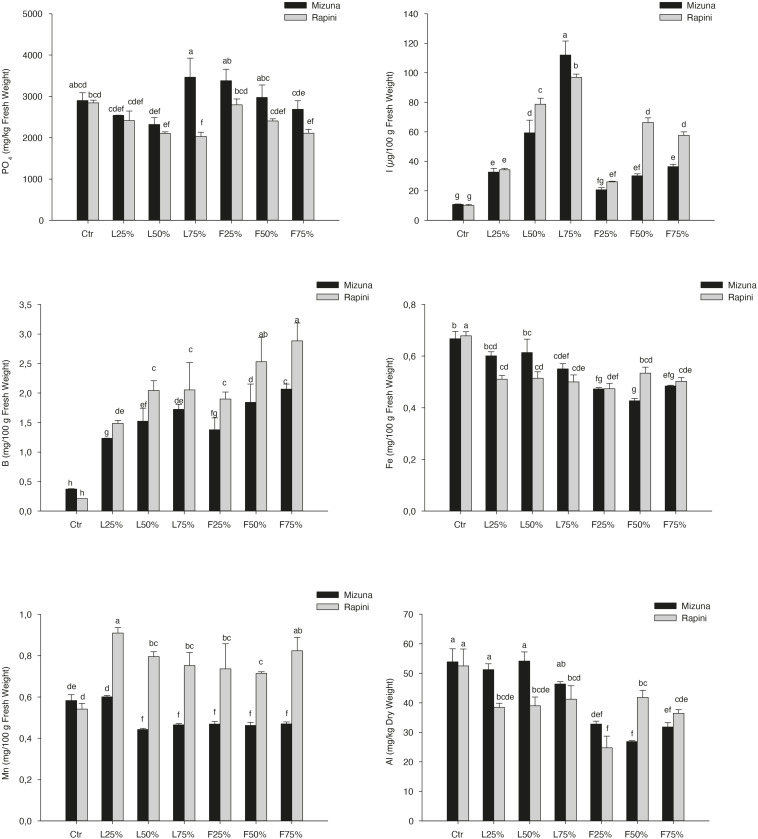
Phosphates, Inorganic iodine, Boron, Iron, Manganese, and Aluminum content in the *Brassica rapa* L. Mizuna and Rapini microgreens as effected by *Posidonia oceanica* (L.) Delile (PO) leaves (L) and fibers (F) in the growing media. CTR (control 100% peat), L_25__%_ (75% peat and 25% PO L), L_50__%_ (50% peat and 50% PO L), L_75__%_ (25% peat and 75% PO L), F_25__%_ (75% peat and 25% PO F), F_50__%_ (50% peat and 50% PO F) and F_75__%_ (25% peat and 75% PO F). Data are expressed as mean ± standard error of treatment (*n* = 3). Significance: ***P ≤ 0.001. Different letters indicate that mean values are significantly different according to the LSD test (α = 0.05).

Yield and DM were influenced by PO residues in the growing media, although to a different extent for Mizuna and Rapini (Table 6). Mizuna plants grown using F_25__%_ showed the highest yield with an increase of 28% compared to peat, while a lower increase was observed with higher percentages of PO F residues in the substrate (Figure 3). In the case of PO L, only a 75% rate allowed a slight increase of the yield with respect to CTR. In Rapini microgreens the highest yield was found in F_25__%_, F_50__%_, L_50__%_ and L_75__%_ (2.92 kg/m^2^, on average), with a 23% increase compared to peat. The highest DM content was observed in Mizuna plants grown in L_75__%_ mixture (18% higher than other treatments), while the PO residues did not affect DW content in Rapini (Figure 3). The mineral composition of macro and microelements in brassica microgreens leaf tissues is reported in Table 7. The K, Zn plant tissues contents were not influenced either by the growing media treatments or by the genotypes, with mean values of 48.4 g/kg and 73.6 mg/kg of DM, respectively. Similarly, growing media composition did not affect SO_4_, although Rapini showed on average higher values than Mizuna. The Ca tissue content was higher in Rapini, on average, and was in general lower when PO residues were used in the mixture, with a mean 28% reduction compared to peat. On the contrary, microgreens showed a higher (23% on average) Mg content compared to peat when PO residues, both L and F, were used at 75% rate, with higher values in Rapini. A similar trend was observed for NO_3_, with a 23% higher content, on average, in L_75__%_ and F_75__%_ compared to peat, although in this case Mizuna showed higher values. As expected, Cl and Na contents were dramatically affected by the presence of PO residues in the growing media, reaching almost double values, in general, at highest PO rates compared to CTR. Rapini showed, on average, higher plant tissue content for both Cl (12%) and Na (13%) compared to Mizuna (Table 7). The presence of PO in the mixtures did not modify the PO_4_ content in Mizuna that was also the genotype with the highest value (2894 mg/kg FW, on average), while in Rapini the highest rate of PO in the growing media reduced the PO_4_ content of 27% compared to the CTR; Figure 4).

**TABLE 6:** Yield and dry weight (DW) of *Brassica rapa* L. Mizuna and Rapini group microgreens, produced in greenhouse, as effected by *Posidonia oceanica* (L.) Delile (PO) leaves (L) and fibers (F) in the growing media.

Treatments	Yield	DW
	kg/m^2^	g/100 g FW
CTR	2.94	6.55
L_25__%_	3.10	6.43
L_50__%_	3.16	5.91
L_75__%_	3.59	6.88
F_25__%_	3.70	6.60
F_50__%_	3.46	6.39
F_75__%_	3.27	6.20
**Genotypes**		
Mizuna	3.86	6.75
Rapini	2.77	6.10
**Significance**		
Treatments (T)	***	*
Genotypes (G)	***	***
T x G	***	*

CTR (control 100% peat), L_25%_ (75% peat and 25% PO L), L_50%_ (50% peat and 50% PO L), L_75%_ (25% peat and 75% PO L), F_25%_ (75% peat and 25% PO F), F_50%_ (50% peat and 50% PO F) and F_75%_ (25% peat and 75% PO F). Data are expressed as mean ± standard error of treatment. FW, fresh weight. Significance: *P ≤ 0.05; ***P ≤ 0.001. Means separation within columns by LSD (α = 0.05).

**TABLE 7:** Mineral composition of *Brassica rapa* L. Mizuna and Rapini group microgreens, produced in greenhouse, as effected by *Posidonia oceanica* (L.) Delile (PO) leaves (L) and fibers (F) in the growing media.

Treatments	K	Ca	Mg	NO_3_	SO_4_	PO_4_	Cl	Na	Al	Zn	I	B	Fe	Mn
					
	g/kg of DW			mg/kg of FW				mg/kg of DW			μg/100 g of FW	mg/100 g of FW		
CTR	52.50	32.37 a	5.07 cd	1740 c	1815	2870	526 d	4596 e	53.19	73.62	10.43	0.29	0.69	0.57
L_25%_	47.02	24.75 bc	4.97 c	1947 ab	1819	2477	687 c	6201 d	44.87	71.89	33.52	1.37	0.56	0.77
L_50%_	48.10	22.47 bc	5.67 abc	1786 c	1729	2211	827 b	8042 c	46.57	77.50	69.03	1.66	0.52	0.58
L_75%_	48.26	21.65 c	5.89 a	2182a	1773	2748	991 a	9090 ab	43.82	72.34	104.5	2.04	0.56	0.66
F_25%_	46.76	25.17 b	4.94 c	1881 bc	1795	3086	651 c	8208 b	28.78	75.99	23.40	1.70	0.49	0.63
F_50%_	47.93	23.85 bc	5.26 abc	1915 bc	1779	2689	830 b	8901 abc	34.37	73.25	48.22	2.20	0.48	0.59
F_75%_	47.86	22.56 bc	5.77 ab	2110 ab	1742	2399	798 b	9759 a	34.12	70.86	47.03	2.41	0.48	0.63
**Genotypes**														
Mizuna	49.73	23.56	4.85	2245	1500	2894	717	7363	42.45	74.52	43.20	1.45	0.55	0.50
Rapini	46.97	25.81	5.89	1630	2057	2386	801	8294	39.19	72.76	52.84	1.87	0.53	0.75
**Significance**														
Treatments(T)	ns	***	*	**	ns	**	***	***	***	ns	***	***	***	***
Genotypes (G)	ns	**	***	***	***	***	**	**	*	ns	***	***	**	***
T x G	ns	ns	ns	ns	ns	*	ns	ns	***	ns	***	***	**	***

CTR (control 100% peat), L_25%_ (75% peat and 25% PO L), L_50%_ (50% peat and 50% PO L), L_75%_ (25% peat and 75% PO L), F_25%_ (75% peat and 25% PO F), F_50%_ (50% peat and 50% PO F) and F_75%_ (25% peat and 75% PO F). Data are expressed as mean ± standard error of treatment. FW, fresh weight; DW, Dry weight. Significance: ns = not significant;*P ≤ 0.05; **P ≤ 0.01; ***P ≤ 0.001. Means separation within columns by LSD (α = 0.05).

On the contrary, I and B concentration exhibited a clear upward trend by increasing PO percentage, both L and F, in the substrate (Table 7 and Figure 4). The highest plant tissue contents of I were found in L_75__%_, with a 936% and 866% increase compared to CTR observed in Mizuna and Rapini, respectively. As a general trend, plants grown in PO F accumulated less I than in PO F. As regard B content, the highest concentration was reached in Rapini plants grown with PO F at 50% e 75% rates (2.71 mg/100 g of FM, on average; Figure 4) corresponding to an increase of almost 13 times compared to CTR. Rapini genotype showed on average a higher B content than Mizuna, except in the case of CTR (0.29 mg/100 g of FM). Adding PO residues in the growing media produced a slight reduction of Fe content in edible part of Rapini (Figure 4) irrespective of percentages used in the mixtures, while in Mizuna the Fe plant tissue concentration was not significantly reduced with PO L at 25% and 50% rates. Mizuna and Rapini microgreens showed an opposite behavior in terms of Mn contents in relation to the PO residues in the growing media. In fact, in Rapini a significantly higher Mn tissue content was found in all plants grown in growing media containing PO residues, with negligible differences in terms of types residues (L or F) and application rate in the substrate (Figure 4). In particular, the highest contents were found in Rapini L_25__%_ and F_75__%_ (0.87 μg/100 g of FW, on average). On the contrary, in Mizuna PO F (50–75% application rate) and PO L (at any rate) reduced the Mn concentration in the leaves (0.46 μg/100 g of FW, on average). The highest values of Al were founded in Mizuna and Rapini CTR, Mizuna L_25__%_, L_50__%_ and L_75__%_ (47.11 mg/kg of DW, on average), while the use of PO F residues at any rate, and of PO L at 75% rate, resulted in a reduction of Al content in edible parts of Mizuna microgreens respect to the CTR, with the lowest values observed in PO F treatments (Figure 4). Similar trend was observed in Rapini microgreens, but in this case a reduced content was found with all PO treatments (Figure 4). The Cr level in Rapini resulted below the limit of quantification (LOQ: 0.6303 μg/l), while in Mizuna microgreens the Cr concentration was not influenced by the presence of PO residues, with a mean value of 0.40 mg/kg of DM (data not show).

**FIGURE 5:**
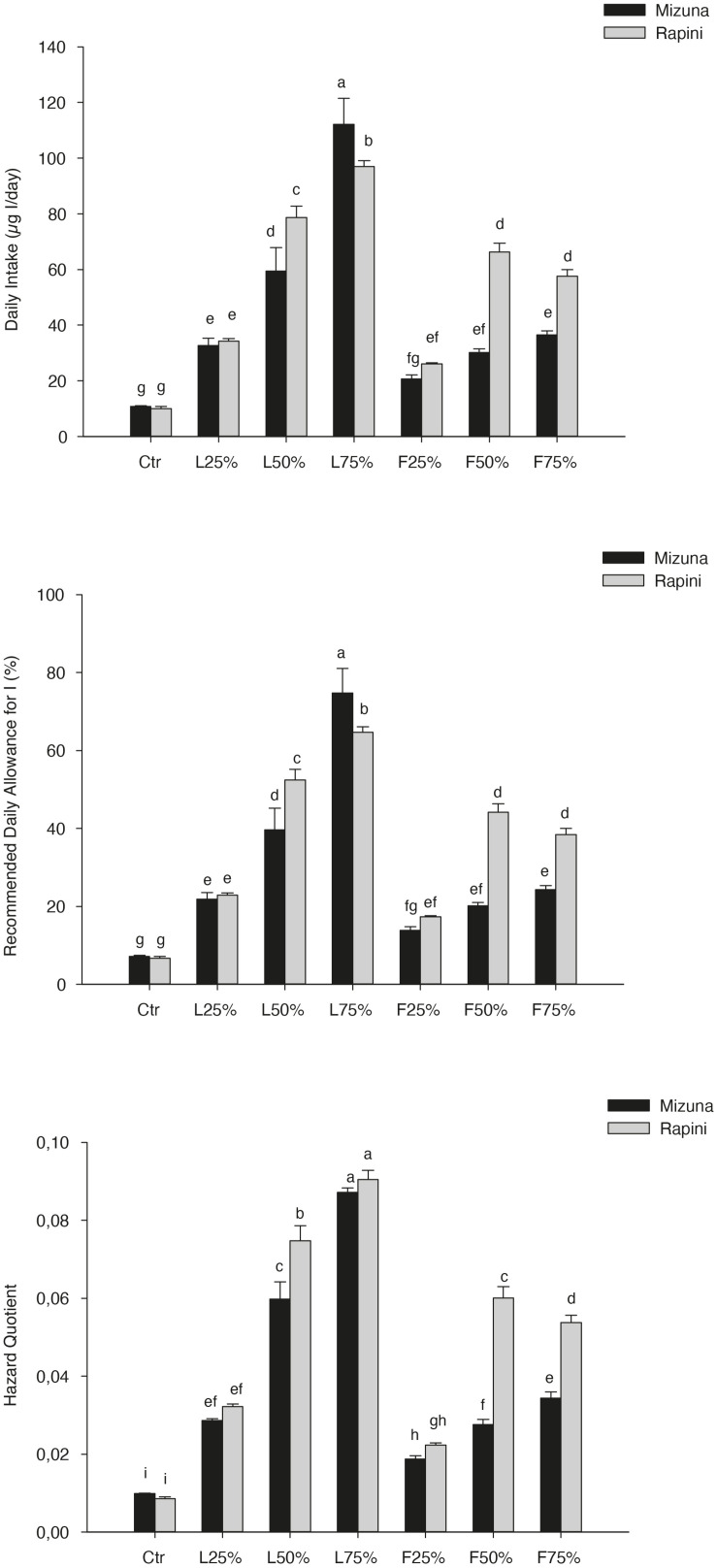
Daily intake, percentage of recommended daily allowance for I (RDA-I) and hazard quotient (HQ) for intake of I through consumption of 100-g portions of Mizuna and Rapini microgreens, produced in greenhouse, by adult humans (70 kg body weight). Significance: ***P ≤ 0.001. Different letters indicate that mean values are significantly different according to the LSD test (α = 0.05).

The Daily intake, percentage of RDA-I and HQ for intake of I through consumption of 100 and 50 g portions of brassica microgreens are reported in Table 8. The application of PO residues significantly increased the values of those parameters, and differences between species were found (Table 8 and Figure 5). The highest values were noted for Mizuna L_75__%_ with the estimated consumption of 100 g of serving size of microgreens. The consumption of both serving sizes of both microgreens species at the highest I content value, does not cover the daily requirement of this micronutrient for adults (150 μg I/day). However, the percentage of RDA-I covered by the consumption of brassica microgreens (both species) cultivated by using PO residues in growing media was higher than CTR. In addition, the consume of 100 g serving size of Mizuna or Rapini microgreens at the highest content of I was characterized by a HQ value lower than 1, which represents a safe dose (Table 8 and Figure 5).

**TABLE 8:** Daily intake, percentage of recommended daily allowance for I (RDA-I) and hazard quotient (HQ) for intake of I through consumption of 100 and 50-g portions of Mizuna and Rapini microgreens, produced in greenhouse, by adult humans (70 kg body weight).

Treatments	100 g Portion of microgreens			50 g Portion of microgreens		
	Daily Intake	RDA-I	HQ_100 g_	Daily Intake	RDA-I	HQ_50 g_
	(μg I/day)	(%)		(μg I/day)	(%)	
CTR	10.43	6.94	0.0092	5.21	3.47	0.0046
L_25%_	33.52	22.34	0.0304	16.76	11.17	0.0152
L_50%_	69.03	46.01	0.0672	34.51	23.09	0.0336
L_75%_	104.5	69.67	0.0888	52.25	34.8	0.0444
F_25%_	23.39	15.59	0.0205	11.69	7.79	0.0103
F_50%_	48.23	32.14	0.0438	24.11	16.07	0.0219
F_75%_	47.04	31.35	0.0441	23.51	15.67	0.0220
**Genotypes**						
Mizuna	43.20	28.78	0.0380	21.59	14.39	0.0190
Rapini	52.85	35.23	0.0489	26.42	17.61	0.0244
Significance						
Treatments (T)	***	***	***	***	***	***
Genotypes (G)	***	***	***	***	***	***
T x G	***	***	***	***	***	***

Data are expressed as mean ± standard error of treatment. Significance: ***P ≤ 0.001. Means separation within columns by LSD (α = 0.05).

**FIGURE 6:**
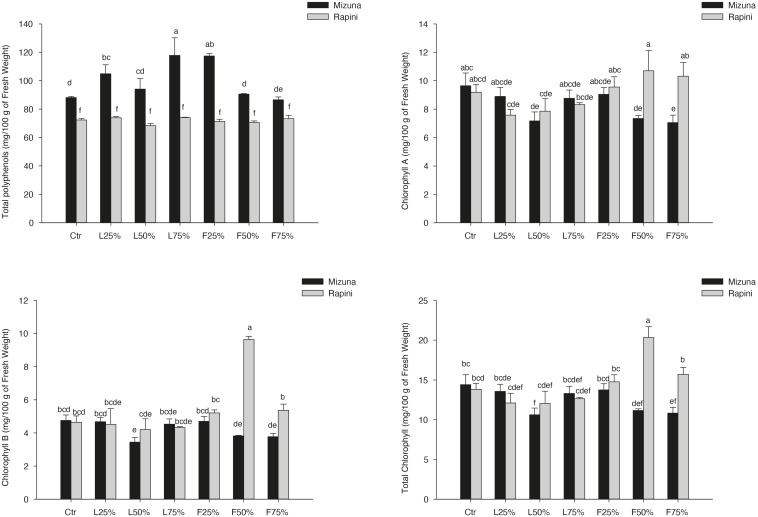
Total polyphenols, chlorophyll A, chlorophyll B and total chlorophyll contents in *Brassica rapa* L. Mizuna and Rapini group microgreens, produced in greenhouse, as effected by *Posidonia oceanica* (L.) Delile (PO) leaves (L) and fibers (F) in the growing media. CTR (control 100% peat), L_25__%_ (75% peat and 25% PO L), L_50__%_ (50% peat and 50% PO L), L_75__%_ (25% peat and 75% PO L), F_25__%_ (75% peat and 25% PO F), F_50__%_ (50% peat and 50% PO F) and F_75__%_ (25% peat and 75% PO F). Significance: **P ≤ 0.01 (Total polyphenols); *P ≤ 0.05 (Chlorophyll A); ***P ≤ 0.001 (Chlorophyll B and Total Chlorophyll). Different letters indicate that mean values are significantly different according to the LSD test (α = 0.05).

The main bioactive compounds (total polyphenols, chlorophyll and carotenoids) measured in microgreens are reported in Table 9 and Figure 6. Total polyphenols, chlorophyll and carotenoids content were influenced by genotype and growing media (Table 9). On average the total polyphenols content was higher in Mizuna than in Rapini. However, while in Mizuna microgreens an increase of total phenols was observed in certain PO treatments, with the highest values in L_25__%_, L_75__%_ and F_25__%_ (113 mg/100 g of FW, on average), in Rapini no differences were observed compared to CTR (72 mg/100 g of FW).

**TABLE 9:** Total polyphenols, chlorophyll and carotenoids contents in *Brassica rapa* L. Mizuna and Rapini group microgreens, produced in greenhouse, as effected by *Posidonia oceanica* (L.) Delile (PO) leaves (L) and fibers (F) in the growing media.

Treatments	Total Polyphenols	CHLa	CHLb	CHLtot	Carotenoids
	
	mg/100 g of FW				
CTR	80.29	9.42	4.70	14.12	4.62 a
L_25__%_	89.44	8.24	4.60	12.83	3.87 bc
L_50__%_	81.23	7.51	3.82	11.34	3.63 c
L_75__%_	95.91	8.54	4.43	12.97	4.35 ab
F_25__%_	94.32	9.29	4.95	14.25	4.62 a
F_50__%_	80.63	9.02	6.72	15.75	3.72 c
F_75__%_	79.91	8.68	4.57	13.26	4.09 abc
**Genotypes**					
Mizuna	99.92	8.27	4.24	12.52	4.19
Rapini	72.00	9.07	5.41	14.49	4.07
**Significance**					
Treatments (T)	***	ns	***	***	**
Genotypes (G)	***	*	***	***	ns
T x G	**	*	***	***	ns

CTR (control 100% peat), L_25%_ (75% peat and 25% PO L), L_50%_ (50% peat and 50% PO L), L_75%_ (25% peat and 75% PO L), F_25%_ (75% peat and 25% PO F), F_50%_ (50% peat and 50% PO F) and F_75%_ (25% peat and 75% PO F). Data are expressed as mean ± standard error of treatment. FW, fresh weight. Significance: ns = not significant;*P ≤ 0.05; **P ≤ 0.01; ***P ≤ 0.001. Means separation within columns by LSD (α = 0.05).

The chlorophyll-A, B and total showed only slight alterations as an effect of treatments. Only CHL-B and CHL-tot showed a relevant increase in Rapini subjected to F_50__%_ treatment (Figure 6). Mizuna showed a trend to lower CHL-A and CHL-tot when PO-F were used at 50% and 75% rates (Figure 6). On average, growing media composition modified the content of carotenoids, with e reduced value in L_25__%_, L_50__%_, F_50__%_ and F_75__%_ treatments compared to CTR (−17%). No differences were observed between genotypes (4.13 mg/100 g of FW, on average).

## Discussion

In the current study we aimed to evaluate the possibility to improve the nutritional quality of products by using not composted PO residues as growing media component, this representing a novelty.

The mixtures resulting from the addition of PO residues presented physical characteristic with values falling in acceptable/optimal range (Abad et al., 2001), and certainly this contributed to preserve, in general, plant growth and thus yield. The PO residues showed a peculiar chemical composition, related to the specific origin of these materials. The high content of Cl and Na are related with the fact that Posidonia is a species adapted to live in the sea, and the residues accumulated on the beach are also exposed to marine aerosol (Voca et al., 2019). Differences have been noted between the two types of residues tested (leaves and fibers). The higher content of N_tot_ in the PO F with respect to PO L is likely due to the partially decomposed state of F. The N_tot_ content measured in peat has to be related mainly with the mineral fertilizers added by the manufacturer, that also influenced NO_3_ and K content, respectively 3.0 and 1.7 times higher in peat than in PO residues, and SO_4_ content as well. Thus, the natural endowment of PO residues in terms of plant nutrients may contribute to reduce the use of mineral fertilizers in substrate-based cropping processes (Mininni et al., 2012). The higher content of I in PO L is probably related to the fact the I is generally stored in greater amount in aerial parts of the plants (Landini et al., 2011). According to Voca et al. (2019), our results confirmed that higher amounts of salts can be found in Posidonia leaf tissues than in the fibers, as an effect of vacuolar ion sequestering and cytosolic osmolyte accumulation. Anyway, the chemical content in our samples resulted quite different compared to the values reported by Cocozza et al. (2011) and Voca et al. (2019), which reported higher contents of K, Mg, Cr, and Mn in PO leaves compared to our data, except for Ca content which resulted higher in our samples. The differences could be due to the washing process applied in our case to PO residues before analysis and subsequent use, in order to allow the removal of salts in excess on the external surface the material, and thus ameliorating its suitability to be used as growing media component. However, B, Fe, and Zn contents were quite in line with values reported by those Authors.

The relatively low DM found in our study could be related to the different senescence stage of the PO leaves compared to what reported by other authors (Voca et al., 2019).

The high concentration of Fe and Mg is related with the physiological peculiarities of seagrass species (Kannan et al., 2011), while the high B content is probably related with the high concentration of this element in seawater (Kabay et al., 2010).

A certain degree of variability in the characteristics of the material should be accepted as normal. In particular, the variability in mineral content composition has been related to the different sites of origin of PO samples, the environmental conditions of growth (namely water pH) and the period of collection (Luy et al., 2012; Scartazza et al., 2017). Therefore, a preliminary chemical characterization of the raw material is advisable in order to be aware of the specific mineral elements endowment.

In general, the high content of certain elements outlines the feasibility of PO residues use as a renewable source of plant nutrients, with particular reference to micro-nutrients endowment and with the related beneficial effects on the nutritional quality of vegetable products.

Higher EC in PO L raw material and PO L based substrates is likely determined by the higher salt accumulation in intact leaves compared to fibers, with particular reference to vacuolar ion sequestering and cytosolic osmolyte accumulation (Touchette, 2007). Mininni et al. (2015) reported similar pH and EC trends in mixtures of peat and Posidonia-based compost.

No phytotoxicity effects on plants were observed in PO treatments, highlighting the possibility of a safe use of PO residues, even without previous composting, as a growing media component. In both experiments conducted in our study the yield was in line with average yield values reported in similar growing conditions (Di Gioia et al., 2017). A slight yield decrease was observed in Mizuna microgreens produced in growth chamber conditions (Experiment 1) on substrates containing PO at high rates, and may probably be attributed to the increase of substrate EC (Pearson correlation, *R*^2^ = −0.807, *P* < 0.000017) (Table 1). However no impairment to plant growth was observed under real greenhouse conditions. The differences in tissue mineral composition of plants grown on different substrates, found in both brassica species and in both experiments, support our hypothesis that the addition of PO residues in growing mixtures may play a role in the nutritional characteristics of products.

In indoor production, the environmental conditions, such as stable mean air temperature, relative humidity, and photosynthetically active radiation (PAR), combined with a higher availability of K in growing media containing PO, has determinated an increase of K tissue concentration in PO L treatment compared to CTR and fibers. The quality of light used in growth chamber may have affected to a certain extent the uptake of macro e micronutrient as reported by different Authors (Brazaitytė et al., 2017; Pinho et al., 2017; Kowalczyk et al., 2020).

On the contrary, in all microgreens produced in this study the Ca plant tissue concentration was reduced by PO in growing media, compared to peat that showed the highest values. Mininni et al. (2015) showed similar results for sweet basil (*Ocimum basilicum*, L.) grown in media based on different percentage of PO compost. In Mizuna and Rapini microgreens this reduction can be related to an increase of Mg content, confirming the antagonism between Ca and Mg. However, it should be noted that in Rapini microgreens only a slight tendency to Mg increase was observed.

The NO_3_ content in vegetables is an important nutritional and consumer health parameter for quality of fresh leafy vegetable products because this anion is listed as an anti-nutritional factor in vegetables (Santamaria, 2006). In fact, as suggested by European Food Safety Authority (European Food Safety Authority (EFSA), 2008) the current acceptable daily intake for this element is 3.7 mg/kg of body weight. In our study, the NO_3_ contents in brassica microgreens plant tissues were, in general, lower respect to limit imposed by Commission Regulation (EU) No 1258/2011 for other leafy vegetables, such as lettuce (3–5 g/kg of FW) and rocket (6–7 g/kg of FW), and to the values reported by Di Gioia and Santamaria (2015) for different brassica microgreens (*Brassica oleracea*, L. var. *italica*, *capitata* and *nipponistica*).

In the indoor production an increasing rate of PO (both L and F) in the growing mixture allowed to reduce the NO_3_ content in edible parts of plant, thus resulting in improved nutritional quality of Mizuna microgreens.

The reduced NO_3_ could be related with the higher I content in growing media and in Mizuna microgreens tissues. Blasco et al. (2010) report similar reduction of NO_3_ in edible parts of lettuce (*Lactuca sativa* L.) after increasing levels of I (20, 40, and 80 μmol/L as KI) in NS, probably related with high phyto-availability of this element. In fact, since the plants absorb this element through ionic channels and transporters of chloride (White and Broadley, 2009), there may occur interference with NO_3_ phyto-availability (Voogt and Jackson, 2010).

Total polyphenols, chlorophyll and carotenoids contents in *Brassica rapa* L. Mizuna and Rapini group microgreens, produced in greenhouse, as effected by *Posidonia oceanica* (L.) Delile (PO) leaves (L) and fibers (F) in the growing media. CTR (control 100% peat),

Conversely, the slight increase of NO_3_ in brassica microgreens produced under greenhouse conditions at high rates of PO residues (L and F) in growing media could be related with a different interaction between treatments and environmental conditions. There are different factors involved in NO_3_ metabolism, such as temperature and light intensity (Santamaria et al., 2001). Probably, under greenhouse natural conditions the positive role of I on reducing NO_3_ content did not take place.

At the same time, the results found in greenhouse production are in agreement with those reported by other authors on Rapini (*Brassica rapa* L.) microgreens (Di Gioia et al., 2017).

The Na content in brassica microgreens produced in PO growing media, was higher respect to our CTR treatment. This result is certainly due to the presence of high concentrations of Na in PO residues (Table 1). Similar results were found in lettuce produced in soilless system by using PO based compost as growing media (Mininni et al., 2012).

Regarding nutritional considerations, the level of Na in CTR treatment (29.2 mg/100 g of FW as average of Mizuna and Rapini) was quite high respect to data reported by United States Department of Agriculture (United States Department of Agriculture (USDA), 2018) for commercial basil microgreens (11 mg/100 g of FW, on average). Notwithstanding this result, the consumption of 100 g of brassica microgreens (F_75__%_) would imply a low intake of this element (Table 4) if compared with the recommended intake which is 2 g Na/day (World Health Organization (WHO), 2014). As regard Al and Cr, elements with potential toxic effects for human health, they are generally present in vegetables at low concentrations (D’Imperio et al., 2018; Rai et al., 2019).

In the indoor production the use of PO residues allowed to further reduce the Cr contents in edible parts of Mizuna microgreens, respect to the CTR. This result could be related with high content of organic and inorganic compounds in PO residue, such as humic acid, Fe-hydroxides and ferrous sulfate which decreases plant uptake due to formation of complexes with different heavy metal such as Cr and As (Campbell, 1995; Warren et al., 2003; Raptis et al., 2018). Similar reduction of Cr was found in sweet basil (*Ocimum basilicum* L.) grown in PO based compost (Mininni et al., 2015).

The reduced Al content in brassica microgreens found in both species could be related with the residue of silicate, generally present in PO (Khiari et al., 2010). In fact, the silicon is able to reduce the uptake of Al (Pontigo et al., 2017).

The I content of plant varies from species to species. An average level of 23.6 μg I/100 g DW of products was reported in leafy vegetables (Haldimann et al., 2005).

The low I found in CTR (100% peat) was quite expected, since on one hand it is documented that seeds generally have extremely low I contents (Herrett et al., 1962; Gonzali et al., 2017), and on the other hand plantlets grown in CTR treatment experienced I-free growing conditions (in both NS and peat substrate used in the experiment, I concentration was < LOQ: 0.1520 μg/l). The increase of I content in brassica microgreens grown in media amended with PO, in particular L, was likely related with the relatively high natural I endowment of the residues. Gonnella et al. (2019) reported the possibility to increase I content in four different Brassica genotypes (broccoli raab, curly kale, mizuna and red mustard) by using a NS enriched with KIO_3_ at different concentrations (5.9 and 11.8 μM of I) in order to improve the nutritional profile of edible plants. In another case (Smolen et al., 2014) an increase of I in edible parts of vegetables was obtained, but it has been reported that in certain cases the I levels might be potentially dangerous, considering that the RDA for adults is 150 μg/day as suggested by World Health Organization (WHO) (2014).

Regarding consumer safety, the Daily intake, the percentage of recommended daily allowance for I (RDA-I) and the hazard quotient (HQ) for intake of I through consumption of 50 and 100 g portions of brassica microgreens, showed low values with respect to other studies (Smoleń et al., 2017). It should be underlined that a defined upper tolerable intake level of iodine concerns only mineral forms of this element. No maximum allowance intake of organically iodine forms has been proposed at the moment.

However, according to our findings, the consume of a 100 g serving size (considered normal for this product) of our brassica microgreens poses no risk to consumer health, contrary to the results reported by Smoleń et al. (2017) reporting that the consumption of 200 g of spinach biofortified with different methods, may compromise the consumer health.

It is worth mentioning that the aim of a biofortification study should not be to obtain products suitable to satisfy the RDA, but possibly to help in filling the dietary gaps (related to I in this case) in specific target groups of population. Excessive concentration of this element in vegetables would pose a risk of excessive iodine intake for humans (the tolerable upper intake level is 1100 μg I/day).

We documented the possibility to improve nutritional profile of brassica microgreens without using iodine chemical fertilizers. On the contrary, we proposed a sustainable approach consisting in the use of a material often managed as a waste.

High increase of B contents was also found in both microgreens species produced by adding PO residues in the growing media (Figures 2, 4), while the B content in CTR brassica microgreens was the lowest, similar to that found in shoot tissues of sprouting broccoli microgreens (Kopsell et al., 2014). This result is correlated with the well documented presence of high concentrations of this element in PO residues (Cocozza et al., 2011) (*Pearson* correlation, *R*^2^ = 0.978, *p* < 0.05 in Mizuna microgreens produced in indoor; *R*^2^ = 0.934, *p* < 0.222 in Mizuna produced in greenhouse; *R*^2^ = 0.874, *p* < 0.008 in Rapini). In general, the application of B in NS increases the tissue content of this element in different parts of vegetables as reported by Ben-Gal and Shani (2002). These Authors reported an increase of B contents in leaves, fruits and stems of tomato after application of different concentrations of this element in irrigation water, in a range of 0.028 – 1.48 mol m^–3^. In our previous study (D’Imperio et al., 2020) the application of B in the NS allowed to increase the B content in commercial and wild genotypes of purslane (*Portulaca oleracea* L.). However, the high increase of B in edible parts of brassica microgreens, in both experiments, did not induce symptoms of plant toxicity, probably related to the typical short growing cycle for microgreens. Similarly, Mininni et al. (2012, 2015) found almost the same B increase trend in lettuce grown in PO-based compost without symptoms of toxicity.

Although generally not considered essential for human health, there are many scientific evidences that B intake within the usual dietary range may influence the metabolism and utilization of Ca and vitamin D in humans, and may have positive effects on bone health (Nielsen, 2008; Hunt, 2012). Sheng et al. (2001) and Nielsen (2018) suggested that for adults an average B intake of 2 mg, on a daily basis, improves bone health. The typical intake of this element is about 1.5 mg/day (European Food Safety Authority (EFSA), 2004). The consume of brassica microgreens grown with PO residue in growing media could allow to improve the intake of this element (Figures 2, 4).

Iron is an essential element for human health: a deficitary intake can induce different chronic and acute effects. 43% of children and 29% of women in reproductive age around the World show different phenomena of anemia, and about 50% of these cases could be the result of iron deficiency (World Health Organization (WHO), 2011). In our study the Fe contents in brassica microgreens were in line with expectations for the same genotype of brassica at the same phenological stage (Xiao et al., 2016).

An impairment to Fe absorption in plants is represented by high pH conditions in the root environment, as the case of the substrates containing PO (Table 2). However, in this study a slight decrease of Fe plant tissue content was observed only in Experiment 2.

On the contrary, Mininni et al. (2012, 2015) found an increase of Fe in lettuce, but not in sweet basil (*Ocimum basilicum* L.), when plants were grown in PO based compost.

Manganese is also an essential element for human health, being a coenzyme in various biochemical processes, and the overall Mn contents in this study were similar to the results found in 30 commercially grown microgreens in Brassicaceae family (Xiao et al., 2016).

Brassica vegetables are known to be rich sources of bioactive compounds, such as glucosinolates, polyphenols, ascorbic acid, carotenoids, and tocopherols, which have human-health effects reportedly involved in preventing cardiovascular diseases and some types of cancers (Cartea et al., 2011; Guzman et al., 2012). In general, an increase of total polyphenols and pigments is associated with plant growing stress (biotic and/or abiotic) conditions (Lattanzio, 2013; Nedbal et al., 2000). In our study the addition of PO in growing media modified somehow the contents of the principal bioactive compounds measured, although it was not possible to identify a clear correlation between the PO rate and the increase of polyphenols and CHL. The overall levels of these compounds was in line with respect to the same brassica microgreens as reported by de la Fuente et al. (2019). The differences between genotypes have to be related with different tolerance to abiotic stress. However, biochemical mechanisms behind the improve of bioactive compounds under salinity eliciting are still not completely understood.

## Conclusion

According to the objective of the study, we demonstrated the possibility to increase the content of some beneficial elements (in particular, I and B) in two different brassica microgreens (Mizuna and Rapini) by using an innovative “ecofriendly” biofortification approach based on the use of PO residues (leaves and fibers) in the growing substrate. This resulted in a source of mineral elements suitable for this scope. In general, the addition of PO in the growing media did not induce phenomena of toxicity neither substantial alteration of plant growth and yield. The microgreens produced with this ecofriendly method showed a high increase of I in edible parts. The HQ calculated underline the safety of these products. Our results support the possibility to produce microgreens with high nutritional profile by recovering an organic material generally treated as a waste, without needs of specific material processing other than crushing and washing. Growing media based on PO residues could allow to reduce the use of peat. Further research is needed to better investigate the physiological mechanisms regulating plant tissue concentrations of beneficial compounds and minerals resulting from the use of the proposed growing media. The effectiveness of the proposed biofortification approach will be tested for the production of other high-health-profile vegetables in soilless conditions, such as baby leafy or fruit vegetables.

## Data Availability Statement

The raw data supporting the conclusions of this article will be made available by the authors, without undue reservation.

## Author Contributions

MD’I and AP made the substantial contributions to the conception or design of the work, performed the analysis of posidonia and microgreens, drafted the work, and did the final approval of the version to be published. FM revised the article critically, drafted the work, and did the final approval of the version to be published. NM revised the article critically and did the final approval of the version to be published. All authors contributed to the article and approved the submitted version.

## Conflict of Interest

The authors declare that the research was conducted in the absence of any commercial or financial relationships that could be construed as a potential conflict of interest.
