# Supplementary material for: Posidonia Natural Residues as Growing Substrate Component: An Ecofriendly Method to Improve Nutritional Profile of Brassica Microgreens
Source: Front Plant Sci. 2021 Jun 24;12:580596. doi: 10.3389/fpls.2021.580596 (PMC8265272; doi:10.3389/fpls.2021.580596)
Supplement: Supplementary file 1 [file Table_1.DOCX]

Supplementary Material

Posidonia natural residues as growing substrate component: an ecofriendly method to improve nutritional profile of brassica microgreens

**Massimiliano D’Imperio^1^, Francesco Fabiano Montesano^1^, Nicola Montemurro^1^ and Angelo Parente^1^***

^1^Institute of Sciences of Food Production, CNR – National Research Council of Italy, Via Amendola 122/D, 70126 Bari, Italy;

**Table 1S**. Concentrations obtained for minerals and NO_3_ determination in NIST-1573a and NRC/SPIN-1(certified reference material).

|  | LOD | LOQ | Certified value and uncertainty | Experimental value and uncertainty |
| --- | --- | --- | --- | --- |
|  | µg/l | | mg/kg of DW | |
| NIST-1573a |  |  |  |  |
| Al | 0.03289 | 0.0996 | 598±7.10 | 575±19.6 |
| B | 0.59262 | 1.7958 | 33.1±0.42 | 32±6.1 |
| Ca | 0.20223 | 0.6128 | 50,450±550 | 50051±2049 |
| Cr | 0.20801 | 0.63034 | 1.988±0.034 | 2.075±0.2913 |
| Fe | 0.81112 | 2.4579 | 367±4.37 | 355±27 |
| K | 15.99416 | 46.467 | 26,760±480 | 25,611±1100 |
| Mg | 0.34969 | 1.0596 | 12000 | 12,758±2949 |
| Na | 0.07113 | 0.2155 | 136±3.70 | 140±19 |
| Mn | 0.74266 | 2.2504 | 246±7.11 | 231±1.6 |
| Zn | 1.45585 | 4.4116 | 30.9±0.55 | 29.9±4.9 |
| I* | 0.05016 | 0.1520 | 0.85 | 0.98±0.05 |
|  |  |  |  |  |
| NRC-SPIN-1 |  |  | mg/g of DM | |
| NO_3_ |  |  | 22.5±0.43 | 22.1±0.60 |

Mg and I: Non-Certified Value. Insufficient information is available to assess the uncertainty associated with the value, and therefore no uncertainty is provided (NIST). *The iodine content was evaluated by using a spectrophotometer method (Gonnella et al., 2019).
